# Longitudinal Changes in Neuroaxonal and Inflammatory CSF Biomarkers in Multiple Sclerosis Patients Undergoing Interferon Beta Therapy

**DOI:** 10.3390/biomedicines13061394

**Published:** 2025-06-06

**Authors:** Simona Petrescu, Maria-Melania Dumitru-Martoiu, Cristina Aura Panea, Charlotte E. Teunissen

**Affiliations:** 1 Department of Neurology, Elias Emergency and University Hospital, 011461 Bucharest, Romania; simona.petrescu@umfcd.ro (S.P.); cristina.panea@umfcd.ro (C.A.P.); 2 Department of Clinical Neurosciences, Carol Davila University of Medicine and Pharmacy, 050474 Bucharest, Romania; 3 Nuffield Department of Clinical Neurosciences, University of Oxford, Oxford OX3 9DU, UK; 4 Neurology Laboratory and Biobank, Department of Clinical Chemistry, VU University Medical Center, 1081 HV Amsterdam, The Netherlands; c.teunissen@amsterdamumc.nl

**Keywords:** multiple sclerosis, biomarkers, cerebrospinal fluid, neurofilament light chain, neurofilament heavy chain, CHI3L1, interferon beta, disease-modifying therapy, longitudinal study

## Abstract

**Background/Objective:** Neurofilament light chain (Nf-L), neurofilament heavy chain (Nf-H), and chitinase 3-like 1 (CHI3L1) are cerebrospinal fluid (CSF) biomarkers of neuroaxonal damage and inflammation in multiple sclerosis (MS). Their longitudinal response to disease-modifying therapies and association with clinical and radiological outcomes remain incompletely understood. The aim of this study is to evaluate the impact of interferon beta (IFN-β) therapy on CSF levels of Nf-L, Nf-H, and CHI3L1 in early relapsing–remitting MS (RRMS) and assess their association with long-term clinical outcomes and MRI activity. **Methods:** We conducted a prospective two-year observational study involving 14 treatment-naive RRMS patients who initiated IFN-β therapy. CSF levels of Nf-L, Nf-H, and CHI3L1 were measured at baseline and after two years. Clinical disability was assessed via the Expanded Disability Status Scale (EDSS) and by studying brain MRI activity. A 15-year clinical follow-up was performed for 12 patients. **Results:** Nf-L levels significantly decreased after two years of IFN-β treatment (*p* = 0.039), while CHI3L1 levels significantly increased (*p* = 0.001). Nf-H levels remained stable. Nf-L and CHI3L1 levels at baseline and follow-up correlated with relapse rate and long-term EDSS. Nf-H levels correlated with EDSS scores but not with relapse or MRI activity. A trend toward a positive correlation between increasing Nf-L levels and MRI activity was observed (*p* = 0.07). **Conclusions:** CSF biomarkers demonstrate differential responses to IFN-β therapy in early RRMS. Nf-L emerges as a sensitive biomarker of treatment response and disease activity, while CHI3L1 may reflect ongoing tissue remodeling and inflammation. These findings support the utility of CSF biomarker monitoring for personalized treatment strategies in MS.

## 1. Introduction

Multiple sclerosis (MS) is an inflammatory neurodegenerative complex disease, characterized by inflammation, demyelination, progressive axonal loss, with a heterogeneous clinical presentation, disease course, pathological, immunological, and radiological features [1].

Inflammation from MS is tightly associated with axonal and neuronal damage [2,3]. Axonal injury can begin in the initial lesion phases, whereas neuronal loss may develop in the progressive phase of the disease [4].

Many biological markers have been studied in MS in order to sub-type the disease, to better quantify disease progression and individual responses to therapy, beyond the clinical and MRI data [5,6].

The study of cerebrospinal fluid (CSF) is of primary importance, as this fluid is most likely to contain traces of biomarkers of the pathological and of reparative processes occurring at the lesion site in MS [7,8,9].

Neurofilaments (NFs) are cytoskeletal components of the neuron providing functional and structural support to axons, stabilizing the axons, determining the axon diameter, and participating in axonal transport [10,11,12]. They are found exclusively within neurons [13,14]. The presence of NFs in CSF reflects neuronal and axonal damage due to the inflammatory process [13,14,15].

Among biomarkers of axonal damage, neurofilaments (NFs) play a major role, representing promising biomarkers for disease progression and for treatment response in MS [2,15].

NFs are composed of different subunits, based on their tail molecular weight: NF-light chain (Nf-L), NF-medium (Nf-M), and heavy chain (Nf-H). Nf-L is a small, soluble protein, and is the most abundant. On the other hand, Nf-H is a larger molecule and more resistant to proteases because it is phosphorylated. Nf-M has not been assessed in studies until now.

When neuronal cells or their axonal membranes are damaged, NFs are released into the interstitial fluid (IF), cerebrospinal fluid (CSF), and blood. Consequently, levels of NFs can serve as biomarkers for monitoring disease progression, treatment response, or drug toxicity [3,16].

Although neurofilament light chain (Nf-L) is not specific to multiple sclerosis (MS) and is elevated in other neurological conditions, it remains the most investigating and promising biomarker for detecting axonal damage in MS and plays a definitive role in predicting disease progression [17,18]. A correlation between CSF Nf-L levels and neuroaxonal injury was found during the entire course of MS [3,19,20]. There is strong evidence that Nf-L levels are elevated in relapsing compared to stable forms of MS, and they can predict future disease progression in terms of relapses, clinical and MRI activity, and treatment response [17]. Nf-L can help stratify patients into those with stable disease and those who do not respond to disease-modifying treatments (DMTs) [17]. Moreover, Nf-L can contribute to a better understanding of the mechanisms underlying demyelination and axonal damage in MS [17,18].

This biological marker must have high sensitivity and specificity and must be analyzed using a reliable laboratory method.

Two methods for measuring Nf-L have been described: electrochemiluminescence (ECL)-based assays and the single-molecule array (Simoa) technology (Quanterix, Billerica, MA, USA) [3,21,22]. ECL was developed for serum Nf-L measurement and offers high sensitivity, a broad dynamic range, and the advantage of requiring only a small sample volume. With the advent of highly sensitive digital enzyme-linked immunoassay (ELISA), also called Single molecule array (Simoa), neurofilaments can now be sensitively quantified in both cerebrospinal fluid (CSF) and blood [22,23,24].

Nf-L levels in CSF and serum are strongly correlated, and patients with relapsing–remitting MS (RRMS) show significantly higher Nf-L concentrations compared to healthy individuals [3,19].

Another biomarker which is thought to have an important role in MS pathology is chitinase 3-like 1 (CHI3L1). Also known as YKL-40, it belongs to the chitin glycoside hydrolase family, a large group of proteins with a prominent role in neuroinflammatory process and tissue remodeling, produced by activated microglia and activated astrocytes following neuronal injury, so its levels in the CSF reflect its endogenous synthesis [25,26]. However, its mechanism of action remains poorly understood, and its role in MS pathogenic mechanisms has not been fully elucidated yet [25]. To support the tissue remodeling effect, it has been shown that higher levels of CHI3L1 are found during remission phases of the disease rather than during relapse phases [25,27]. Elevated levels of CHI3L1 have been detected in the CSF of patients with clinically isolated syndrome (CIS) and MS [28,29]. CHI3L1 can be a potential indicator for diagnosing and monitoring the disease stage and treatment response in MS, higher levels having prognostic implications [30,31].

## 2. Materials and Methods

Inflammation and neurodegeneration are central components of multiple sclerosis (MS) pathology. Recent studies have shown that biomarkers of neuroaxonal damage are detectable even in the early stages of MS.

Given the known response of neurofilament light chain (Nf-L) and CHI3L1 to anti-inflammatory treatment, we hypothesized that these markers might also be influenced by interferon beta therapy.

Our aim was to assess whether neuroaxonal biomarkers such as Nf-L and Nf-H, as well as the inflammatory and tissue remodeling biomarker CHI3L1, found in cerebrospinal fluid (CSF), are affected by immunomodulatory treatment with interferon beta (IFN-β), and whether their levels correlate with treatment response, as measured by clinical scales and brain MRI.

To this end, we conducted a two-year observational study on a small cohort of patients with early relapsing–remitting MS. The study was conducted between 2009 and 2011. We analyzed CSF levels of neuroaxonal and inflammatory biomarkers before and after two years of IFN-β treatment. All patients were treatment-naive at baseline, having received no prior immunomodulatory or immunosuppressive therapy, a factor that makes this cohort particularly distinctive.

An additional clinical assessment was conducted 15 years after the baseline point.

### 2.1. Methods

#### 2.1.1. Study Population

We studied 14 patients diagnosed with relapsing–remitting multiple sclerosis (RRMS) according to the revised 2005 McDonald criteria [32]. Cerebrospinal fluid (CSF) was collected from all patients at baseline, and from 13 of them after two years of follow-up.

All patients provided written informed consent to participate in the study, which was approved by the Local Ethics Committee (P121108 CLE) and conducted in accordance with the principles outlined in the Declaration of Helsinki (1975, revised in 2013). At the time of enrollment, all patients were treatment-naive with respect to immunomodulatory therapies and had been diagnosed with MS within the previous 12 months. All patients were in clinical remission, with no recent relapse. Following the baseline evaluation, all patients began treatment with interferon beta (IFN-β) products.

At both baseline and follow-up, patients underwent clinical assessment using the Expanded Disability Status Scale (EDSS) [33], and brain Magnetic Resonance Imaging (MRI) was performed. The EDSS evaluations were consistently conducted by the same neurologist trained in its administration. Additionally, EDSS was assessed 15 years after baseline for twelve patients, as one patient had left the program. The assessments were part of their annual follow-up, and the data were collected from patient records in the National Program for Multiple Sclerosis.

All patients were interviewed and asked about relapses in the follow-up period. A relapse was defined as the appearance of a new symptom or the worsening of a pre-existing symptom attributable to MS, lasting ≥24 h, occurring without fever, accompanied by a new neurological abnormality, and preceded by a period of stability or improvement lasting ≥30 days [34]. Pseudo-relapses due to infections or elevated body temperature were not included in the analysis.

MRI of the brain with gadolinium was performed according to the MS protocol using a 1.5 Tesla scanner (Signa HDx, GE Healthcare, Milwaukee, WI, USA). T2-weighted and contrast-enhanced T1-weighted axial slices were obtained at all visits, with a slice thickness of 10 mm. An MRI was considered active if at least one enhancing lesion was identified. At each time point, EDSS assessment and MRI were performed prior to cerebrospinal fluid (CSF) collection. The CSF samples were collected at least three months after completion of intravenous methylprednisolone treatment and only from patients who were clinically stable and in remission at the time of lumbar puncture.

#### 2.1.2. CSF Analysis

CSF samples were collected at each time point, at baseline and after two years of treatment. Following standard procedures, the samples were centrifuged and stored at −70 °C. All samples were analyzed in a single session. Levels of Nf-L, Nf-H, and CHI3L1 in CSF were measured at baseline and after two years of treatment with IFN-β, and these were correlated with treatment response. All biomarkers were analyzed using commercially available ELISA kits (R&D Systems, Minneapolis, MN, USA).

#### 2.1.3. Statistical Analysis

We performed statistical analysis with Statistical Package for the Social Sciences (version 27.0, SPSS Inc., Chicago, IL, USA). The level of statistical significance was *p* < 0.05 (two-tailed). We performed Wilcoxon Signed Rank test for small groups to assess the longitudinal dynamics of variables within the same group. We performed correlations to test the associations between clinical–EDSS, relapse rate and MRI metrics with Nf-L, Nf-H, and CHI3L1 levels, using the Spearman correlations when the variables considered were numeric or Fisher’s exact method when variables considered were nonparametric, due to the small cohort size.

## 3. Results

### 3.1. Patients’ Characteristics

Our population had a medium age of 32.14 (23–47) years, the majority were female (10 out of 14). Medium EDSS at baseline was 2.32 (1.0–4.5), and 2.39 (0.0–5.5) after two years of treatment. After 15 years the EDSS increased, the medium EDSS being 4.45 (2.0–7.5). Only two patients showed clinically active disease during the first year of follow-up (one of whom experienced a relapse), and for them we decided to escalate therapy after the two-year treatment period. Additionally, only five patients showed clinically active disease after the two-year follow-up (including one relapse during the entire follow-up period).

Demographic, clinical, and paraclinical (MRI) data are show in Table 1 and Table 2. CSF data for the 13 patients who accepted two successive lumbar punctures is shown in Table 3.

### 3.2. The Impact of the Immunomodulatory Treatment with IFN Beta on Biomarkers in CSF

**Figure 1:**
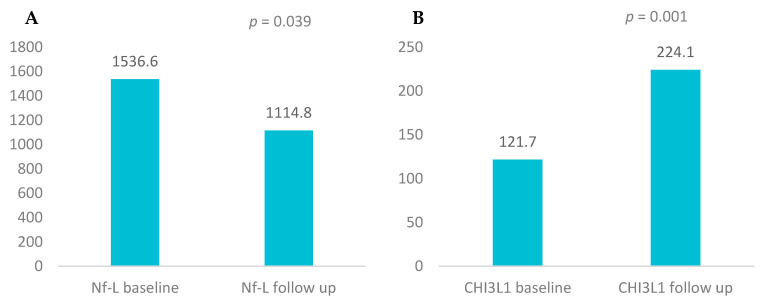
(**A**) Mean levels of Nf-L baseline/follow-up, which declined significantly under INF beta therapy (Wilcoxon Signed Rank test, *p* = 0.039) and (**B**) mean levels of CHI3L1 baseline/follow-up, which increased significantly under INF beta therapy (Wilcoxon Signed Rank test, *p* = 0.001).

We analyzed the mean values of Nf-L, Nf-H, and CHI3L1 over the two-year treatment period with IFN-β using the Wilcoxon Signed Rank test (Table 3; Figure 1). One patient who did not undergo the second lumbar puncture was excluded from the analysis.

The mean Nf-L concentration significantly decreased from 1536.6 ± 987.9 ng/mL at baseline to 1114.8 ± 1092.8 ng/mL after 2 years. In contrast, Nf-H concentrations showed a non-significant decrease from 539.1 ± 373 ng/mL to 573.8 ± 480.8 ng/mL. CHI3L1 levels increased markedly over the 2-year period, rising from 121.7 ± 74.7 ng/mL at baseline to 224.1 ± 143 ng/mL, indicating a statistically significant elevation.

Longitudinal variation in these biomarkers under IFN treatment was significant for Nf-L (*p* = 0.039) and highly significant for CHI3L1 (*p =* 0.001), but not for Nf-H (*p* = 0.34).

While Nf-L levels declined significantly and CHI3L1 levels increased significantly, Nf-H levels remained relatively stable.

Immunomodulatory treatment with interferon beta (IFN-β) resulted in a marked reduction in Nf-L levels and a significant increase in CHI3L1 levels in the CSF.

### 3.3. CSF Biomarkers and Demographic Data

In the overall group, Nf-L levels (baseline *p* = 0.558 and follow-up *p* = 0.362), CHI3L1 levels (baseline *p* = 0.139 and follow-up *p* = 0.634), and Nf-H levels at follow-up (*p* = 0.775) did not correlate with patient age. Only Nf-H levels at baseline showed a significant correlation with age (*p* = 0.016).

### 3.4. CSF Biomarkers and EDSS

**Figure 2:**
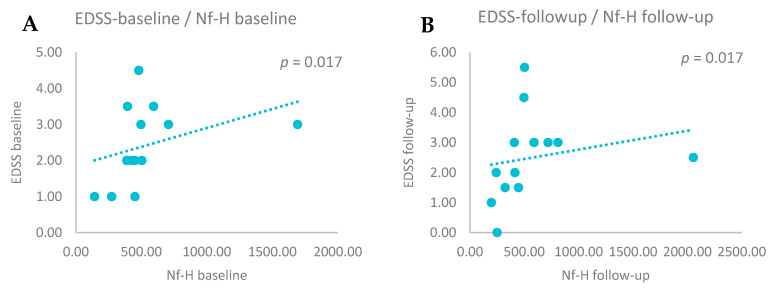
(**A**) Positive correlations between EDSS baseline and Nf-H baseline levels (Spearman correlation, *p* = 0.017). (**B**) Positive correlations between EDSS follow-up and Nf-H follow-up levels (Spearman correlation, *p* = 0.017).

To assess the relationship between EDSS and CSF biomarker levels, we performed correlation analyses. A positive correlation was observed between EDSS scores and Nf-H levels at baseline (r = 0.624, *p* = 0.017) and after two years (r = 0.645, *p* = 0.017)—Figure 2. No significant correlations were found for Nf-L (baseline *p* = 0.436 and follow-up *p* = 0.101) or CHI3L1 (baseline *p* = 0.318 and follow-up *p* = 0.1).

**Figure 3:**
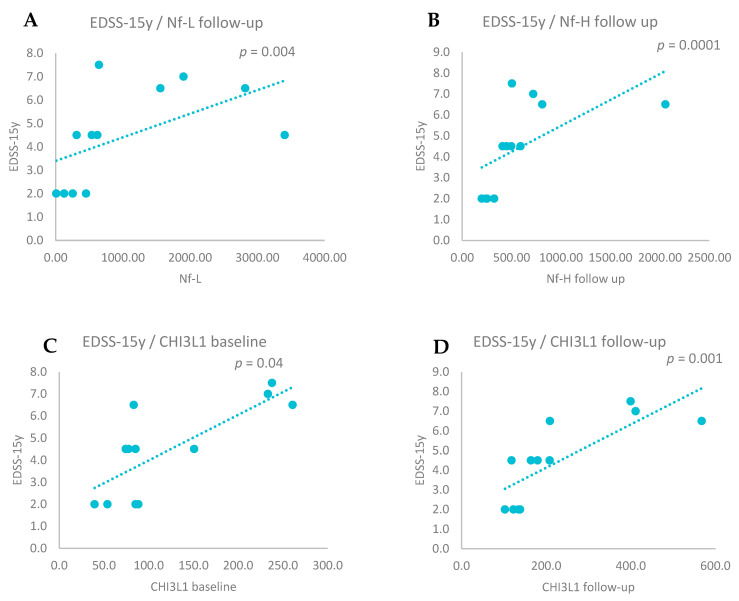
(**A**) Positive correlations between EDSS measured after 15 years and Nf-L follow-up levels (Spearman correlation, *p* = 0.004). (**B**) Positive correlations between EDSS measured after 15 years and Nf-H follow-up levels (Spearman correlation, *p* = 0.0001). (**C**) Positive correlations between EDSS measured after 15 years and CHI3L1 baseline levels (Spearman correlation, *p* = 0.04). (**D**) Positive correlations between EDSS measured after 15 years and CHI3L1 follow-up levels (Spearman correlation, *p* = 0.001).

Positive correlations were observed between EDSS scores from the 15-year point and Nf-L levels (r = 0.763, *p* = 0.004), Nf-H levels (r = 0.857, *p* = 0.0001) (both measured at follow-up), and CHI3L1 levels measured at baseline (r = 0.650, *p* = 0.04) and follow-up (r = 0.839, *p* = 0.001)—Figure 3.

There was a tendency for positive correlations between EDSS score from the 15-year point and levels of Nf-L (r = 0. 430, *p* = 0.07) and Nf-H (r = 0.425, *p* = 0.06), both measured at baseline.

We assessed clinical evolution through changes in EDSS and evaluated whether it correlated with variations in CSF biomarkers: light and heavy neurofilament proteins (Nf-L, Nf-H) and chitinase 3-like 1 (CHI3L1). We assigned a value of 0 if the clinical (EDSS) and paraclinical (Nf and CHI3L1) data decreased or remained stable, and a value of 1 if their values increased. Analyzing EDSS variation, eleven patients (86.4%) had stable or improved EDSS scores throughout the entire treatment period with IFN-β. Among these clinically stable patients, up to seven (53.8%) showed decreased Nf-L levels, and up to six (46%) showed decreased Nf-H levels. CHI3L1 levels remained consistently elevated at the follow-up point in all patients.

After fifteen years, EDSS scores had increased in eleven patients out of twelve (91.6%), with three of them (25%) showing increased Nf-L and Nf-H levels at the follow-up point (after two years of INF therapy).

We performed a Fisher’s exact analysis and found no significant association between EDSS variation and changes in Nf-L (χ^2^ = 1.051, *p* = 0.462) or Nf-H (χ^2^ = 1.477, *p* = 0.359) over the follow-up period. Since CHI3L1 levels were consistently elevated at follow-up in all patients, statistical analysis was not feasible.

### 3.5. CSF Biomarkers and Relapse Rate

**Figure 4:**
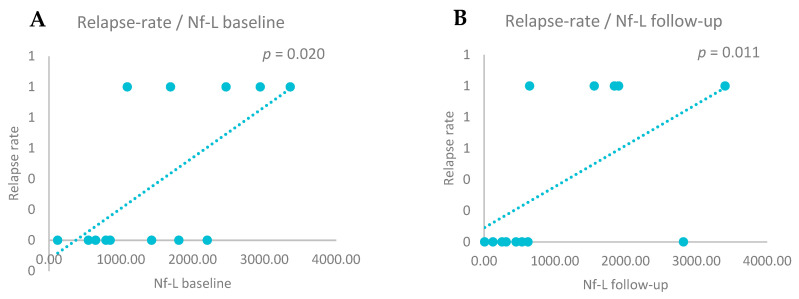
(**A**) Positive correlations between relapse rate and Nf-L baseline levels (Spearman correlation, *p* = 0.020). (**B**) Positive correlations between relapse rate and Nf-L follow-up levels (Spearman correlation, *p* = 0.011).

**Figure 5:**
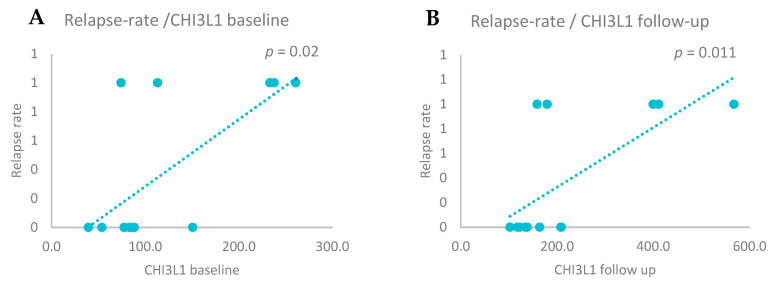
(**A**) Positive correlations between relapse rate and CHI3L1 baseline levels (Spearman correlation, *p* = 0.020. (**B**) Positive correlations between relapse rate and CHI3L1 follow-up levels (Spearman correlation, *p* = 0.011).

We also assessed the relationship between relapses and CSF biomarker levels by performing statistical correlations. A positive correlation was observed between relapse rate and Nf-L levels at baseline (r = 0.634, *p* = 0.020) and after two years (r = 0.675, *p* = 0.011)*,* and between relapse rate and CHI3L1 at baseline (r = 0.684, *p* = 0.010) and after two years (r = 0.634, *p* = 0.020)—Figure 4 and Figure 5. No significant correlations were found for Nf-H (baseline *p* = 0.326 and follow-up *p* = 0.259).

### 3.6. CSF Biomarkers’ Variation and Radiological Signs of Disease Activity on Brain MRI

We looked at MRI activity after two years of treatment and checked if it correlated with the variance of light and heavy neurofilaments (Nf-L, Nf-H) proteins and with chitinase 3-like protein 1 in CSF.

Five patients (38.5%) had at least one Gd (+) lesion on the MRI at baseline and follow-up time points.

We considered as 0, if the variance of NFs and CHI3L1 decreased or stayed stable and as 1 if this value increased. For the brain MRI we considered a non-active MRI (without gadolinium enhancing lesions) as 0 and an active MRI (with at least one gadolinium enhancing lesion) as 1.

We performed a Fischer’s exact analysis test, and we observed a tendency for positive correlation (χ^2^ = 3.25; *p* = 0.07) between MRI activity with the presence of at least one gadolinium enhancing lesion, and Nf-L variance with an increased level after two years, but not for Nf-H (χ^2^ = 1.59; *p* = 0.2) or for CHI3L1.

**Figure 6:**
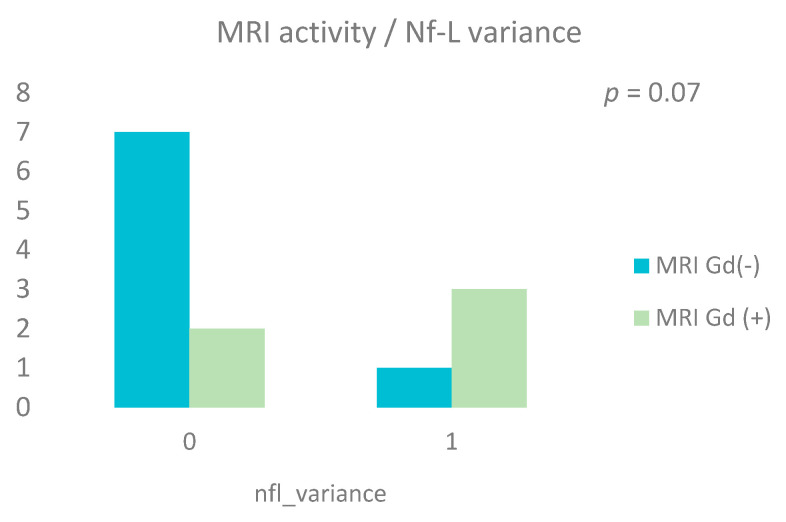
NF-L variance and MRI activity during the follow-up period (chi square analyses test, χ^2^ = 3.25, and *p* = 0.07).

Contrast-enhancing lesions showed a positive correlation with Nf-L values in CSF, but not with Nf-H—Figure 6.

## 4. Discussion

In this longitudinal study of 14 patients with recently diagnosed relapsing–remitting multiple sclerosis (RRMS), we evaluated the dynamics of cerebrospinal fluid (CSF) biomarkers, neurofilament light chain (Nf-L), neurofilament heavy chain (Nf-H), and chitinase 3-like protein 1 (CHI3L1) in response to a two-year period of interferon-beta (IFN-β) therapy. Our findings suggest differential behavior in these biomarkers under treatment, reflecting distinct underlying pathophysiological processes.

A significant decrease in Nf-L concentrations over the two-year treatment period and an association between higher Nf-L levels and increased relapse rate highlight its potential as a responsive biomarker of neuroaxonal damage, disease activity, and therapeutic efficacy in MS. This agrees with prior studies linking Nf-L levels to acute axonal injury and inflammatory disease activity, which reinforces the potential of Nf-L as a dynamic biomarker for monitoring inflammatory disease burden [15,35,36].

Furthermore, our data suggested a trend toward increased Nf-L in patients with ongoing MRI activity, though this did not reach statistical significance (*p* = 0.07). This underscores the role of Nf-L as a sensitive indicator of radiological disease progression, even in clinically stable patients and reinforces the potential of Nf-L as a dynamic marker of inflammatory disease activity, even in small cohorts, and suggests that more extensive studies may confirm this relationship.

Beyond multiple sclerosis, Nf-L and CHI3L1 have emerged as important biomarkers in various neurological disorders. Elevated Nf-L levels have been documented in a range of neurodegenerative and acute neurological conditions, including Alzheimer’s disease, amyotrophic lateral sclerosis (ALS), frontotemporal dementia, traumatic brain injury, and stroke, where they reflect active axonal injury and correlate with disease severity and progression [16,21]. Similarly, CHI3L1 has been associated with neuroinflammation and glial activation in diseases such as Alzheimer’s disease, Parkinson’s disease, traumatic brain injury, and HIV-associated neurocognitive disorders, highlighting its role in chronic inflammatory and neurodegenerative processes beyond MS. These cross-disease associations underline the biological relevance of these markers and support their use in assessing diverse forms of central nervous system pathology. Their application in MS may, therefore, not only inform disease-specific activity but also provide insights into broader neuroinflammatory and neurodegenerative mechanisms [28].

Recent prospective studies evaluating tissue damage markers in the CSF of patients with relapsing–remitting MS, both prior to initiation of various therapies and after variable follow-up periods, have demonstrated a significant reduction in Nf-L levels [37,38]. These findings are consistent with our results, further supporting the role of Nf-L as a reliable biomarker of acute axonal injury driven by inflammatory processes [39]. While the decrease in Nf-L levels was modest in magnitude, it was statistically significant, suggesting that even first-line immunomodulatory therapy can modulate axonal injury in the early disease course.

In contrast, Nf-H concentrations did not change significantly during treatment yet demonstrated strong correlations with disability scores (EDSS) at baseline, follow-up, and even 15 years later. This supports the previous literature identifying Nf-H as a marker of cumulative axonal damage, which may be less responsive to short-term changes but a strong predictor of long-term disability progression [40]. Because of its lower molecular weight and lower phosphorylation rate, Nf-L may diffuse earlier to CSF than Nf-H. The observed stability of Nf-H levels may reflect either a plateau in neurodegeneration under therapy or a slower turnover rate compared to Nf-L [41,42].

One of the most intriguing findings of our study is the consistent and significant increase in CSF of CHI3L1 concentrations after two years of treatment, independently of clinical or radiological disease activity. Our findings, showing increased CSF CHI3L1 after IFN-β initiation, differ from prior CSF based results. We hypothesize that the compartment (CSF vs. serum), timing, or disease activity at baseline may explain this discrepancy. It is possible that persistent subclinical inflammation within the CNS is reflected in CSF but not peripheral CHI3L1 dynamics.

The increase in CHI3L1 concentrations in CSF suggests an association with chronic glial activation and chronic inflammation in progressive forms of MS [25,43]. The consistent increase across patients, including those with stable clinical and radiological status, suggests that CHI3L1 may reflect subclinical or smoldering neuroinflammatory activity that persists despite the disease control, processes not captured by clinical or radiological measures [29,44]. The correlations of CHI3L1 and Nf-L with relapses rates further highlight their potential as prognostic markers for ongoing inflammatory burden [43,45].

Clinically, most patients exhibited stable or improved EDSS scores during the initial two years of treatment, consistent with reductions or stabilization in Nf-L and Nf-H levels. However, at 15 years, EDSS scores had increased in most patients, and early biomarker profiles appeared predictive of long-term disability. Notably, higher levels of Nf-L, Nf-H, and CHI3L1 at two years correlated with worse disability outcomes at 15 years, underscoring their prognostic value. This suggests that early biomarker profiles might have prognostic utility in identifying patients at higher risk for long-term disability, even when short-term clinical improvement is observed.

Notably, we found no statistically significant associations between changes in EDSS and CSF biomarker variations, possibly due to the relatively stable clinical course observed in most patients and the limited sample size. However, the association of baseline and follow-up Nf-L and CHI3L1 with relapse frequency and baseline and follow-up Nf-H with EDSS supports their role in prognostic stratification [26,29,45].

To our knowledge, this is a unique prospective study to monitor neuroaxonal and inflammatory markers in the CSF of MS patients who were treatment-naive at baseline. The extended clinical follow-up over 15 years provides valuable insight into the long-term relevance of early biomarker changes.

This study has limitations, including the small cohort size and lack of a control group, which reduce statistical power and generalizability, increase the risk of Type II error, and may lead to overinterpretation of trends with marginal significance (e.g., *p* = 0.07). The analyses were exploratory in nature and not corrected for multiple comparisons due to the small sample size.

The absence of an untreated comparator group prevents definitive attribution of biomarker changes to IFN-β therapy alone. Future studies should aim to include untreated or alternatively treated control groups to better delineate treatment-specific effects from natural disease progression.

Additionally, the absence of longitudinal serum biomarker data also restricts our ability to compare peripheral and central nervous system dynamics. Nevertheless, the paired CSF analyses and uniform treatment protocol strengthen the internal consistency of our findings.

Despite the small sample size, the consistency of our findings with previously published studies adds confidence to the validity of our results [46,47]. The novelty of our study lies in the use of longitudinal CSF biomarker data (rather than serum or plasma), a 15-year clinical follow-up, and the evaluation of treatment-naive patients initiating IFN-β therapy. We emphasize that early combined CSF measurements of Nf-L and CHI3L1 may help identify patients less likely to respond to IFN-β, thereby contributing to more personalized treatment strategies in MS.

## 5. Conclusions

This study reinforces the clinical utility of CSF biomarkers in RRMS. Nf-L serves as a sensitive marker of treatment response and disease activity, while Nf-H appears more closely related to cumulative neuroaxonal damage and clinical disability. CHI3L1 may reflect ongoing glial activation and subclinical inflammation that persist despite apparent disease stability.

Importantly, higher Nf-L, Nf-H, and CHI3L1 levels after two years of therapy were associated with greater disability at 15 years, suggesting that early CSF biomarker profiles may have prognostic utility. These findings reinforce the potential value of integrating CSF biomarkers into early disease monitoring to optimize therapeutic strategies.

Together, these biomarkers may offer complementary insights into MS pathophysiology and therapeutic monitoring. Further studies in larger, diverse cohorts are needed to validate these findings and explore their potential for personalized treatment strategies.

## Figures and Tables

**Table 1:** Demographic and clinical data.

	N	Min	Max	Mean	Std. Deviation	Median (lq, hq)	Std. Error of Mean
Sex: no (%)	13						
Female	9 (69.2%)	n/a	n/a	n/a	n/a	n/a	n/a
Male	4 (30.8%)	n/a	n/a	n/a	n/a	n/a	n/a
Age	13	23	47	32.54	8.800	30 (24.5, 41.5)	2.44
EDSS baseline	13	1.0	4.5	2.43	1.0963	2.0 (1.5, 3.25)	0.3041
EDSS follow-up	13	0.0	5.5	2.5	1.4434	2.5 (1.5, 3.0)	0.4003
EDSS 15y	12	2.0	7.0	4.45	2.0939	4.5 (2.0, 6.5)	0.6045

Abbreviations: n/a—not applicable, EDSS—Expanded Disability Status Scale, lq—lower quartile, and hq—highest quartile.

**Table 2:** Brain MRI.

	N
Brain MRI baseline: no (%)	
Gd (−) lesions	8 (61.5%)
Gd (+) lesions	5 (38.5%)
Brain MRI follow-up: no (%)	
Gd (−) lesions	8 (61.5%)
Gd (+) lesions	5 (38.5%)

Abbreviations: MRI—Magnetic Resonance Imaging and Gd—gadolinium.

**Table 3:** CSF data.

	N	Min	Max	Mean	Std. Deviation	Median (lq, hq)	Std. Error of Mean
Nf-L (pg/mL):							
Baseline	13	122	3362	1536.6	987.9	1430 (723, 2337)	274
Follow-up	13	9	3408	1114.8	1092.8	621 (284, 1873)	303.1
Nf-H (pg/mL):							
Baseline	13	147.7	1693.9	539.1	373	451.6 (391.6, 548.8)	103.4
Follow-up	13	197.8	2056.8	573.8	480.8	447.2 (287.2, 654.6)	133.3
CHI3L1 (ng/mL):							
Baseline	13	39.4	260.8	121.7	74.1	85.3 (75.9, 191.9)	20.7
Follow-up	13	102.4	567.4	224.1	143	163.9 (128.7, 304.1)	39.6

Abbreviations: Nf-L—neurofilament with light chain, Nf-H—neurofilament with heavy chain, and CHI3L1—chitinase 3-like 1.

## Data Availability

The data used to support the findings of this study are included within the article.

## References

[1] Lassmann H. (2018). Multiple sclerosis pathology. Cold Spring Harb. Perspect. Med..

[2] Franciotta D. (2004). Biological markers in multiple sclerosis. Int. MS J..

[3] Kouchaki E., Dashti F., Mirazimi S.M.A., Alirezaei Z., Jafari S.H., Hamblin M.R., Mirzaei H. (2021). Neurofilament light chain as a biomarker for diagnosis of multiple sclerosis. EXCLI J..

[4] Kuhlmann T., Moccia M., Coetzee T., Cohen J.A., Correale J., Graves J., Marrie R.A., Montalban X., Yong V.W., Thompson A.J. (2022). Time for a new mechanism-driven framework to define multiple sclerosis progression. Lancet Neurol..

[5] Yang J., Hamade M., Wu Q., Wang Q., Axtell R., Giri S., Mao-Draayer Y. (2022). Current and Future Biomarkers in Multiple Sclerosis. Int. J. Mol. Sci..

[6] Costa J., Macaron G., Khaled K.J.A. (2023). Biomarkers in multiple sclerosis: An update. Biomark. Neuropsychiatry.

[7] Berger T., Reindl M. (2006). Biomarkers in Multiple Sclerosis: Role of Antibodies. Dis. Markers.

[8] Teunissen C.E., Khalil M. (2012). Neurofilaments as biomarkers in multiple sclerosis. Mult. Scler. J..

[9] Teunissen C., Menge T., Altintas A., Álvarez-Cermeño J.C., Bertolotto A., Berven F.S., Brundin L., Comabella M., Degn M., Deisenhammer F. (2013). Consensus definitions and application guidelines for control groups in cerebrospinal fluid biomarker studies in multiple sclerosis. Mult. Scler. J..

[10] Khalil M., Teunissen C.E., Otto M., Piehl F., Sormani M.P., Gattringer T., Barro C., Kappos L., Comabella M., Fazekas F. (2018). Neurofilaments as biomarkers in neurological disorders. Nat. Rev. Neurol..

[11] Uphaus T., Bittner S., Gröschel S., Steffen F., Muthuraman M., Wasser K., Weber-Krüger M., Zipp F., Wachter R., Gröschel K. (2019). NfL (Neurofilament Light Chain) Levels as a Predictive Marker for Long-Term Outcome After Ischemic Stroke. Stroke.

[12] Moseby-Knappe M., Mattsson N., Nielsen N., Zetterberg H., Blennow K., Dankiewicz J., Dragancea I., Friberg H., Lilja G., Insel P.S. (2019). Serum Neurofilament Light Chain for Prognosis of Outcome after Cardiac Arrest. JAMA Neurol..

[13] Gresle M.M., Shaw G., Jarrott B., Alexandrou E.N., Friedhuber A., Kilpatrick T.J., Butzkueven H. (2008). Validation of a novel biomarker for acute axonal injury in experimental autoimmune encephalomyelitis. J. Neurosci. Res..

[14] Yuan A., Rao M.V., Veeranna N.R., Nixon R.A. (2017). Neurofilaments and Neurofilament Proteins in Health and Disease. Cold Spring Harb. Perspect. Biol..

[15] Bittner S., Oh J., Havrdová E.K., Tintoré M., Zipp F. (2021). The potential of serum neurofilament as biomarker for multiple sclerosis. Brain.

[16] Lee Y., Lee B.H., Yip W., Chou P., Yip B.-S. (2020). Neurofilament Proteins as Prognostic Biomarkers in Neurological Disorders. Curr. Pharm. Des..

[17] Ghezzi A., Neuteboom R.F. (2023). Neurofilament Light Chain in Adult and Pediatric Multiple Sclerosis: A Promising Biomarker to Better Characterize Disease Activity and Personalize MS Treatment. Neurol. Ther..

[18] Gaetani L., Blennow K., Calabresi P., Di Filippo M., Parnetti L., Zetterberg H. (2019). Neurofilament light chain as a biomarker in neurological disorders. J. Neurol. Neurosurg. Psychiatry.

[19] Kuhle J., Kropshofer H., Haering D.A., Kundu U., Meinert R., Barro C., Dahlke F., Tomic D., Leppert D., Kappos L. (2019). Blood neurofilament light chain as a biomarker of MS disease activity and treatment response. Neurology.

[20] Bridel C., Van Wieringen W.N., Zetterberg H., Tijms B.M., Teunissen C.E., the NFL Group (2019). Diagnostic Value of Cerebrospinal Fluid Neurofilament Light Protein in Neurology: A Systematic Review and Meta-analysis. JAMA Neurol..

[21] Delaby C., Bousiges O., Bouvier D., Fillée C., Fourier A., Mondésert E., Nezry N., Omar S., Quadrio I., Rucheton B. (2022). Neurofilaments contribution in clinic: State of the art. Front. Aging Neurosci..

[22] Petzold A., Altintas A., Andreoni L., Bartos A., Berthele A., Blankenstein M.A., Buee L., Castellazzi M., Cepok S., Comabella M. (2010). Neurofilament ELISA validation. J. Immunol. Methods.

[23] Wilson D.H., Rissin D.M., Kan C.W., Fournier D.R., Piech T., Campbell T.G., Meyer R.E., Fishburn M.W., Cabrera C., Patel P.P. (2016). The Simoa HD-1 Analyzer: A Novel Fully Automated Digital Immunoassay Analyzer with Single-Molecule Sensitivity and Multiplexing. J. Lab. Autom..

[24] Kuhle J., Barro C., Andreasson U., Derfuss T., Lindberg R., Sandelius Å., Liman V., Norgren N., Blennow K., Zetterberg H. (2016). Comparison of three analytical platforms for quantification of the neurofilament light chain in blood samples: ELISA, electrochemiluminescence immunoassay and Simoa. Clin. Chem. Lab. Med..

[25] Floro S., Carandini T., Pietroboni A.M., De Riz M.A., Scarpini E., Galimberti D. (2022). Role of Chitinase 3-like 1 as a Biomarker in Multiple Sclerosis: A Systematic Review and Meta-analysis. Neurol. Neuroimmunol. Neuroinflamm..

[26] Jatczak-Pawlik I., Jurewicz A., Domowicz M., Ewiak-Paszyńska A., Stasiołek M. (2024). CHI3L1 in Multiple Sclerosis—From Bench to Clinic. Cells.

[27] Lucchini M., De Arcangelis V., Piro G., Nociti V., Bianco A., De Fino C., Di Sante G., Ria F., Calabresi P., Mirabella M. (2023). CSF CXCL13 and Chitinase 3-like-1 Levels Predict Disease Course in Relapsing Multiple Sclerosis. Mol. Neurobiol..

[28] Hrabar D., Bakula D., Vrkljan N., Ratkajec V., Glavcic G., Miler M., Pelajic S., Rogic D., Blazevic N., Pavic T. (2023). YKL-40 as a biomarker in various inflammatory diseases: A review. Biochem. Medica.

[29] Talaat F., Abdelatty S., Ragaie C., Dahshan A. (2023). Chitinase-3-like 1-protein in CSF: A novel biomarker for progression in patients with multiple sclerosis. Neurol. Sci..

[30] Mohammed M.S., Al-Rubae’I S.H., Rheima A.M., Al-Kazazz F.F. (2024). A novel sandwich ELISA method for quantifying CHI3L1 in blood serum and cerebrospinal fluid multiple sclerosis patients using sustainable photo-irradiated zero-valence gold nanoparticles. Results Chem..

[31] Cubas-Núñez L., Gil-Perotín S., Castillo-Villalba J., López V., Tarazona L.S., Gasqué-Rubio R., Carratalá-Boscá S., Alcalá-Vicente C., Pérez-Miralles F., Lassmann H. (2021). Potential Role of CHI3L1+ Astrocytes in Progression in MS. Neurol. Neuroimmunol. Neuroinflamm..

[32] Polman C.H., Reingold S.C., Edan G., Filippi M., Hartung H.-P., Kappos L., Lublin F.D., Metz L.M., McFarland H.F., O’Connor P.W. (2005). Diagnostic criteria for multiple sclerosis: 2005 Revisions to the ‘McDonald Criteria’. Ann. Neurol..

[33] Kurtzke J.F. (1983). Rating neurologic impairment in multiple sclerosis: An expanded disability status scale (EDSS). Neurology.

[34] Thompson A.J., Banwell B.L., Barkhof F., Carroll W.M., Coetzee T., Comi G., Correale J., Fazekas F., Filippi M., Freedman M.S. (2018). Diagnosis of multiple sclerosis: 2017 revisions of the McDonald criteria. Lancet Neurol..

[35] Yik J.T., Becquart P., Gill J., Petkau J., Traboulsee A., Carruthers R., Kolind S.H., Devonshire V., Sayao A.-L., Schabas A. (2022). Serum neurofilament light chain correlates with myelin and axonal magnetic resonance imaging markers in multiple sclerosis. Mult. Scler. Relat. Disord..

[36] Bosch A.v.D., Fransen N., Mason M., Rozemuller A.J., Teunissen C., Smolders J., Huitinga I. (2022). Neurofilament Light Chain Levels in Multiple Sclerosis Correlate With Lesions Containing Foamy Macrophages and With Acute Axonal Damage. Neurol. Neuroimmunol. Neuroinflamm..

[37] Rosenstein I., Axelsson M., Novakova L., Blennow K., Zetterberg H., Lycke J. (2021). Exploring CSF neurofilament light as a biomarker for MS in clinical practice; a retrospective registry-based study. Mult. Scler. J..

[38] Delcoigne B., Manouchehrinia A., Barro C., Benkert P., Michalak Z., Kappos L., Leppert D., Tsai J.A., Plavina T., Kieseier B.C. (2020). Blood neurofilament light levels segregate treatment effects in multiple sclerosis. Neurology.

[39] Benkert P., Meier S., Schaedelin S., Manouchehrinia A., Yaldizli Ö., Maceski A., Oechtering J., Achtnichts L., Conen D., Derfuss T. (2022). Serum neurofilament light chain for individual prognostication of disease activity in people with multiple sclerosis: A retrospective modelling and validation study. Lancet Neurol..

[40] Petzold A., Eikelenboom M.J., Keir G., Grant D., Lazeron R.H.C., Polman C.H., Uitdehaag B.M.J., Thompson E.J., Giovannoni G. (2005). Axonal damage accumulates in the progressive phase of multiple sclerosis: Three year follow up study. J. Neurol. Neurosurg. Psychiatry.

[41] Şanlı E.Ş., Tüzün E., Akbayır E., Türkoğlu R. (2021). Phosphorylated neurofilament heavy chain (pNFH) in clinically isolated syndrome and multiple sclerosis. Noro Psikiyatr. Arsivi.

[42] De Angelis F., Ammoscato F., Parker R.A., Plantone D., Doshi A., John N.A., Williams T., Stutters J., MacManus D., Schmierer K. (2025). Neurofilament heavy chain in secondary progressive multiple sclerosis. Mult. Scler. J..

[43] Gil-Perotin S., Castillo-Villalba J., Cubas-Nuñez L., Gasque R., Hervas D., Gomez-Mateu J., Alcala C., Perez-Miralles F., Gascon F., Dominguez J.A. (2019). Combined Cerebrospinal Fluid Neurofilament Light Chain Protein and Chitinase-3 Like-1 Levels in Defining Disease Course and Prognosis in Multiple Sclerosis. Front. Neurol..

[44] Ahmad I., Wergeland S., Oveland E., Bø L. (2023). An Association of Chitinase-3 Like-Protein-1 With Neuronal Deterioration in Multiple Sclerosis. ASN Neuro.

[45] Sellebjerg F., Royen L., Sørensen P.S., Oturai A.B., Jensen P.E.H. (2018). Prognostic value of cerebrospinal fluid neurofilament light chain and chitinase-3-like-1 in newly diagnosed patients with multiple sclerosis. Mult. Scler. J..

[46] Di Filippo M., Gaetani L., Centonze D., Hegen H., Kuhle J., Teunissen C.E., Tintoré M., Villar L.M., Willemse E.A., Zetterberg H. (2024). Fluid biomarkers in multiple sclerosis: From current to future applications. Lancet Reg. Health Eur..

[47] Magliozzi R., Cross A.H. (2020). Can CSF biomarkers predict future MS disease activity and severity?. Mult. Scler. J..

